# RhoC Regulates the Proliferation of Gastric Cancer Cells through Interaction with IQGAP1

**DOI:** 10.1371/journal.pone.0048917

**Published:** 2012-11-07

**Authors:** Yan Wu, Yan Tao, Yongchang Chen, Wenrong Xu

**Affiliations:** School of Medical Science and Laboratory Medicine, Jiangsu University, Zhenjiang, Jiangsu, China; Innsbruck Medical University, Austria

## Abstract

Our previous research results showed that both Ras homolog family member C (RhoC) and IQ-domain GTPase-activating protein 1 (IQGAP1) were over-expressed in gastric cancer tissues and cells, but their role in tumorigenensis has not been addressed clearly. Herein we reported the proliferation stimulating effect of RhoC and IQGAP1 on gastric cancer cells and the interaction between two proteins in regulating the proliferation of gastric cancer cells. Plasmids and viral constructs encoding target siRNA and DNA were used to alter the expression of RhoC and IQGAP1. MTT method and BrdU incorporation assay were used for analyzing the effect of RhoC and different structures of IQGAP1 on proliferation. Protein levels of IQGAP1 and RhoC in cell lines were detected by Western blotting. Immunofluorescence and Co-Immunoprecipitation assays were applied to investigate the localization and binding between RhoC and IQGAP1. The results showed that RhoC, IQGAP1 and the C-terminal fragment of IQGAP1 significantly stimulated the proliferation of gastric cancer cells, and enhanced the expression of cyclin E and cyclin D1. By contrast, reduction of endogenous IQGAP1 or RhoC by siRNA attenuated cell proliferation. The depletion of IQGAP1 expression by siRNA significantly blocked the proliferative activity of constitutively active RhoC, while RhoC silencing by siRNA had no effect on IQGAP1-induced proliferation in gastric cancer cells. Co-immunoprecipitation and Immunofluorescence assays showed that RhoC and IQGAP1 bound each other. In conclusion, our results suggest that RhoC stimulates the proliferation of gastric cancer cells through recruiting IQGAP1 as an effector.

## Introduction

Rho GTPases can induce certain intracellular signal transduction and impact various cellular activities, including reorganization of the actin cytoskeleton, gene transcription, survival, and proliferation [1], [2]. Although three Rho isoforms, RhoA, RhoB, and RhoC, exhibit more than 85% amino acid identity, they confer differences in function in cells [3]. RhoA and RhoC proteins have been shown to have a positive role in proliferation and malignant transformation [4], [5], [6], whereas the role of RhoB in these processes appears to be more divergent [7], [8]. Most of the studies showed that RhoC had multiple functions in tumor metastasis, orchestrating the action of multiple downstream effectors, degradation and reconstruction of the extracellular matrix (ECM). However, there remains some controversy about the role of RhoC in regulating cell proliferation [9], [10], [11], [12]. The results from Pile laboratory indicated that RhoA and RhoC siRNA represent powerful tools for inhibiting cancer cell proliferation, invasiveness, and angiogenesis both *in vitro* and *in vivo* [9]. Sun *et al* found that intratumoral injection of RhoA or RhoC siRNA to nude mice can inhibit tumor growth [13]. In contrast, Faried *et al* reported that RhoA promotes tumor growth more than RhoC, whereas RhoC induces distant metastasis in comparison to RhoA [11], in agreement with those observations by Clark and colleagues [14]. A study by Ikoma and colleagues showed that RhoC did not affect tumor growth but enhances the metastatic nature of lung cancer by stimulating cell motility [10]. Therefore, further study is needed to identify the role of RhoC in regulating biological activities of tumor cells.

IQ-domain GTPase-activating proteins (IQGAPs) are important regulators of the cellular processes that include cytoskeletal rearrangements in cell migration, proliferation and cytokinesis [15], [16], [17]. IQGAP1 is one of the members of the three related mammalian IQGAPs and changing in intracellular IQGAP1 expression or function has reported to alter cell activities [17], [18], [19], [20]. As a scaffold protein, IQGAP1 binds multiple proteins, such as oncogenes β-catenin and Src, tumor suppressor E-cadherin and the Rho GTPases Cdc42 and Rac1, and causes the alteration of cellular behaviors, especially for cancer cells. Our previous study showed that the higher expressions of RhoC and IQGAP1 in gastric cancer tissue were significantly reversely correlated with the differentiation of the gastric cancer cells [21]. Moreover, the protein levels were also relevant to the proliferation and migration of the cancer cells. Therefore, we hypothesize that both RhoC and IQGAP1 may play important role in cancer cell transformation and proliferation and there is a potential association between them. In the present research, we studied the influence of RhoC and different structures of IQGAP1 on cancer cell proliferation and cell cycle related proteins. We also investigated the relationship between the two molecules by joint application of siRNA interference and viral infection technique.

**Figure 1:**
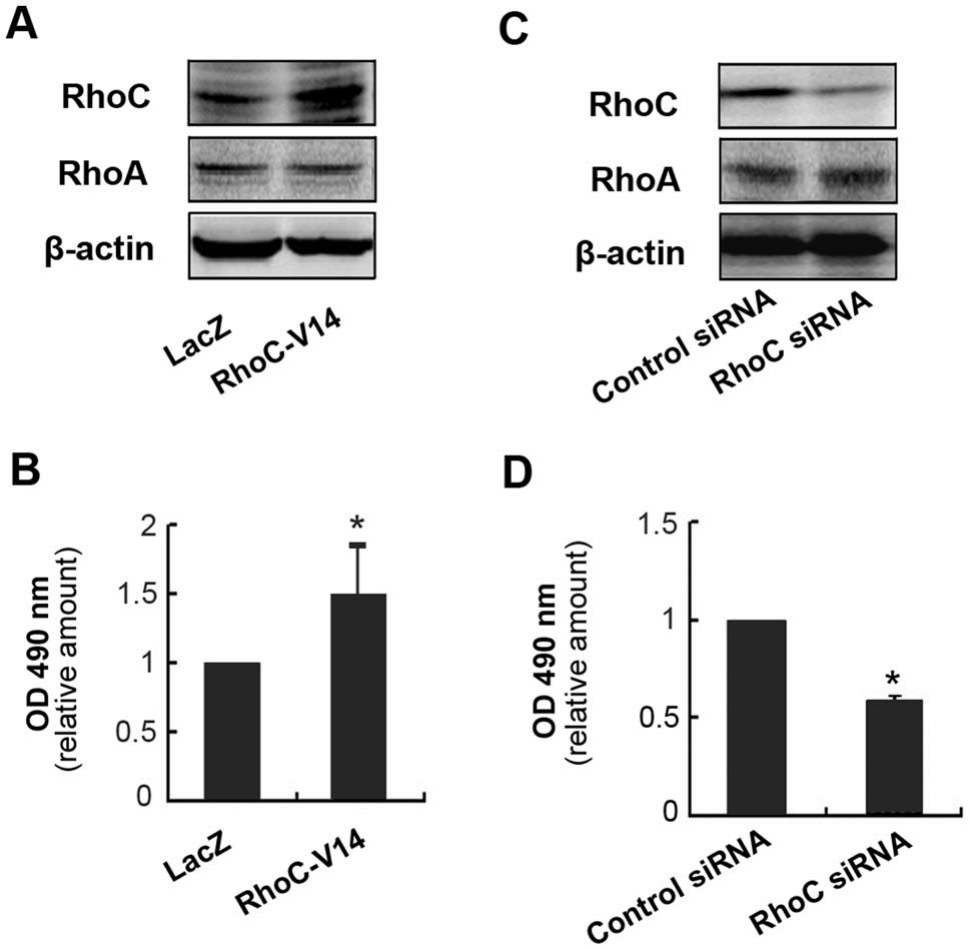
The effect of RhoC on proliferation of BGC-823 cells. (**A**) Western blot analysis of RhoC expression in BGC-823 cells infected with Ad-RhoC-V14. (**B**) The relative proliferation activity of BGC-823 cells infected with Ad-RhoC-V14 was examined by MTT assay. (*P<0.05, compared to Ad-LacZ group). (**C**) The protein expression of RhoC in BGC-823 cells transfected with RhoC siRNA. (**D**) Knocking down of RhoC inhibited proliferation of BGC-823 cells (MTT assay, *P<0.05, compared to Control siRNA group). The data are the means ± SD from three independent experiments each performed in duplicate.

## Materials and Methods

### Reagents

The gastric cancer cell lines BGC-823 [22] and African green monkey kidney fibroblast cell lines COS-7 were provided by the Institute of Cell Biology (Shanghai, China). The Green Fluorescent Protein (GFP) plasmid and cell transfection reagent Lipofectamin™ 2000 were from Invitrogen (Carlsbad, CA); The plasmids encoding Flag tagged IQGAP1 (pFlag-IQGAP1), Flag tagged IQGAP1 C-terminal fragment (pFlag-IQGAP1-C), and Flag tagged IQGAP1 N-terminal fragment (pFlag-IQGAP1-N), and adenoviral vectors encoding β-galactosidase (pAd-LacZ), a constitutively active form of RhoC (pAd-RhoC-V14), full length IQGAP1 (pAd-IQGAP1), and the C-terminal fragment of IQGAP1 (pAd-IQGAP1-C) were kind gifts from Dr. Gerry Boss and Dr. Renate Pilz in University of California, San Diego, USA. Human-EGF, BrdU, Mouse anti-BrdU antibody, DNase I, MTT, Hoechst 33342, FITC- and Cy3-conjugated secondary antibodies were from Sigma (St. Louis, MO). The anti-IQGAP1 antibody (against N-terminal fragment, 314–422 aa), anti-RhoA antibody, anti-IgG antibody, anti-CDK1/CDK2 antibody, short-interfering RNAs (siRNA) for RhoC and negative control were from Santa Cruz Biotechnology (Santa Cruz, CA). The Rho C siRNA is a pool of 3 target-specific 20–25 nt siRNAs designed to knock down the gene expression with the sequences as follows: sequence1, sense 5′-CUACUGUCUUUGAGAACUAtt-3′and antisense 5′-UAGUUCUCAA- AGACAGUAGtt-3′; sequence2, sense 5′-GCAGGAAGACUAUGAUCGAtt-3′ and antisense 5′-UCGAUCAUAGUCUUCCUGCtt-3′; sequence3, sense 5′-CAC- ACCAGCACUUUAUACAtt-3′ and antisense 5′-UGUAUAAAGUGCUGGUGUG- tt-3′. The anti-IQGAP1 antibody (against C-terminal fragment corresponding to amino acids 863–1657 of human IQGAP1) was from Millipore (Billerica, MA). The anti-RhoC antibody was from Abcam (Cambridge, MA). The anti-cyclin B antibody was from Bioworld Technology (St. Louis Park, MN). The anti-cyclin D1 antibody and anti-cyclin E antibody were from Boster (Wuhan, China). The siRNA for IQGAP1 was synthesized by Qiagen (Valencia, CA), the target sequence was IQGAP1 5′-AAGTTCTACGGGAAGTAATTG-3′ (5058–5078 bp). Horseradish peroxidase (HRP)-conjugated secondary antibody was from Rockland Immunochemicals (Gilbertsville, PA). Protein G Plus/Protein A-Agarose was from Calbiochem (San Diego, CA). Electrochemiluminescence (ECL) reagents were from Millipore (Billerica, MA). All reagents used in this study were of analytical grade.

**Figure 2:**
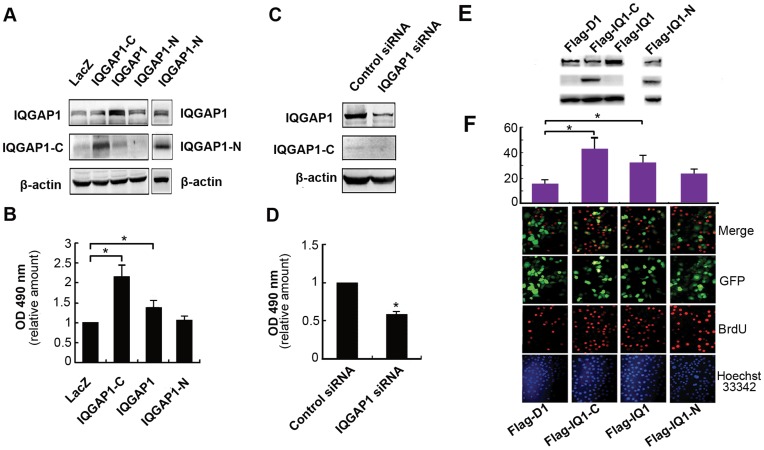
The effect of IQGAP1 on proliferation of BGC-823 cells. (**A**) Western blot analysis of IQGAP1 expression in gastric cancer cell BGC-823 lines infected with Ad-LacZ, Ad-IQGAP1-C, Ad-IQGAP1-N or Ad-IQGAP1. (**B**) In the MTT assay, IQGAP1-C and IQGAP1 over expression cells both have more proliferation activity than control group (*P<0.05, compared to Ad-LacZ group). (**C**) The protein expression level of IQGAP1 in gastric cancer cell line BGC-823 transfected with IQGAP1 siRNA. (**D**) The proliferation of BGC-823 cells transfected with IQGAP1 siRNA were examined by MTT assay. (*P<0.05, compared to Control siRNA group). (**E**) BGC-823 cells were transiently transfected with plasmids Flag-IQGAP1, Flag-IQGAP1-C, or Flag-IQGAP1-N for 48 h. Western blotting showed the expression of IQGAP1, IQGAP1-N, and IQGAP1-C constructs in BGC-823 cell lines. Equal amounts of cell lysate from each group were loaded and blotted with anti-IQGAP1 antibodies (against C-terminal fragment or N- terminal fragment). (**F**) BrdU assay was used for analysis cell proliferation. Representative images of BGC-823 cells expressing the indicated IQGAP1 constructs were stained with antibodies against BrdU (second panels red) and Hoechst 33342 for nuclei (first panel, blue). The percentage of cells with BrdU incorporation was calculated. The mean ± SD of three independent experiments is presented (*P<0.05).

### Cell Culture, Transfection and Infection

BGC-823 and COS-7 cells were cultured in 1×Dulbecco’s Modified Eagle’s medium (DMEM) supplemented with 10% fetal bovine serum (FBS) at 37°C in 5% CO_2_ atmosphere. The medium was changed every second day and the cells were sub-cultured at confluence. For transfection, the cells were sub-cultured the day before the process and the transfection of gastric cancer cells with plasmids or siRNA was performed according to the manufacturer’s instruction. For infection, 293A cells were transfected with adenoviral vectors pAd-LacZ, pAd-RhoC-V14, pAd-IQGAP1 and pAd-IQGAP1-C, and the viral particles produced by the cells were harvested and amplified. The viruses were named Ad-LacZ, Ad-RhoC-14, Ad-IQGAP1 and Ad-IQGAP1-C respectively and were used to infect BGC-823 cells. On the day before the infection, BGC-823 cells were freshly seeded at 70–80% confluence and the infection process was performed according to the manufacturer’s instruction on second day.

**Figure 3:**
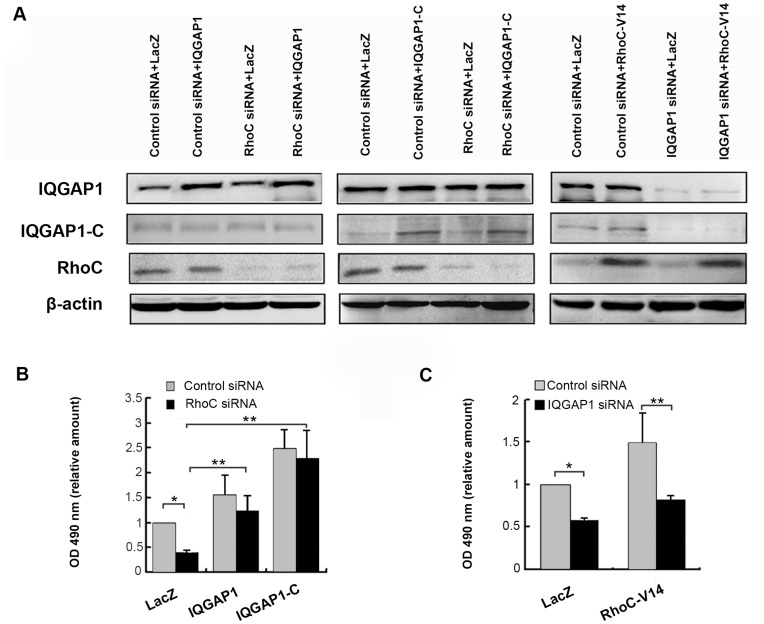
The proliferation-stimulating effect of RhoC was blocked by IQGAP1 siRNA. (**A**) The protein expression levels of IQGAP1, IQGAP1-C and RhoC in BGC-823 cells. BGC-823 cells were transiently transfected with IQGAP1 siRNA or RhoC siRNA for 24 h. The transfected cells were afterwards infected with Ad-RhoC-V14, Ad-IQGAP1-C or Ad-IQGAP1 for additional 48 h followed by Western blotting. (**B**) RhoC depletion did not significantly affect IQGAP1 or IQGAP1-C induced proliferation of BGC-823 cells. (**C**) The silencing of IQGAP1 by siRNA markedly inhibited the RhoC-induced cell proliferation in BGC-823 cells. (MTT assay, *P<0.05; **P<0.01). The data are the means ± SD from three independent experiments each performed in duplicate.

### Western Blotting

Cultured cells were washed three times in PBS and lysed with RIPA buffer (50 mM Tris-HCl, pH 7.4, 1% (v/v) Triton X-100, 1 mM EDTA, 1 mM leupeptin, 1 mM phenylmethylsulfonyl fluoride, 10 mM NaF, 1 mM Na_3_VO_4_). Equal amounts of protein were separated by 8% or 12% SDS–PAGE according to the protein molecular weight**.** The primary antibodies were incubated over night at 4°C, and the corresponding secondary antibodies were incubated for 1 h at RT. Three washes were performed with TBS/T after each antibody incubation. ECL reagents were used to show the positive bands on the membrane.

**Figure 4:**
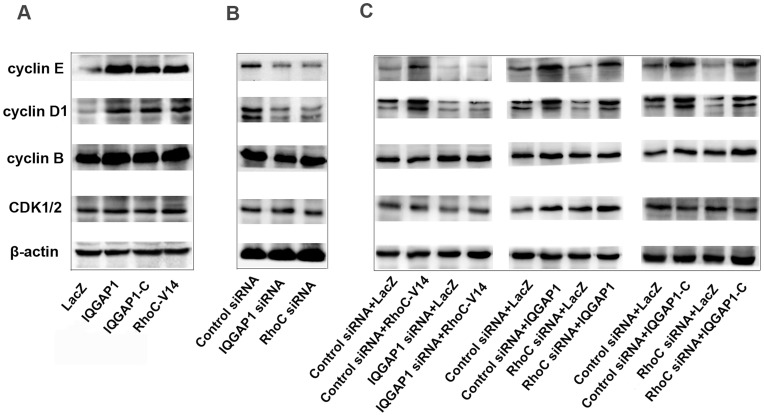
The effects of RhoC and IQGAP1 on expression of cell cycle-related proteins. (**A**) BGC-823 cells were infected with Ad-IQGAP1, Ad-IQGAP1-C or Ad-RhoC-V14 for 48 h, and Western blot was used to analyze the expressions of cyclin E, cyclin D1 cyclin B and CDK. (**B**) BGC-823 cells were transfected with IQGAP1 siRNA or RhoC siRNA for 72 h, and the expressions of cyclin E, cyclin D1, cyclin B and CDK were analyzed by Western blotting. (**C**) The protein expressions of cyclin E, cyclin D1, cyclin B and CDK in BGC-823 cells which were transiently transfected with IQGAP1 siRNA or RhoC siRNA for 24 h and afterwards infected with Ad-RhoC-V14, Ad-IQGAP1-C or Ad-IQGAP1 for additional 48 h (Results of Western blotting).

### MTT Assay

0.5–1×10^3^cells in 150 µl of medium were plated in the well of 96-well plates. After attachment, the cells were infected with corresponding adenovirus for 48 h, or transfected with siRNA for 72 h, respectively. In combination groups, cells were transfected with siRNA targeting RhoC or IQGAP1 for 24 h and then infected with Ad-IQGAP1-C/Ad-IQGAP1 or Ad-RhoC-V14 for an additional 48 h at 37°C in 5% CO_2_. The cultured cells were washed with PBS, treated with 20 µl of MTT (0.5 mg/ml), and then incubated at 37°C for 1 h. The medium was removed and 100 µl of dimethylsulfoxide (DMSO) were added to each well. The absorbance was determined at 490 nm using microplate reader. All assays were performed in triplicate.

**Figure 5:**
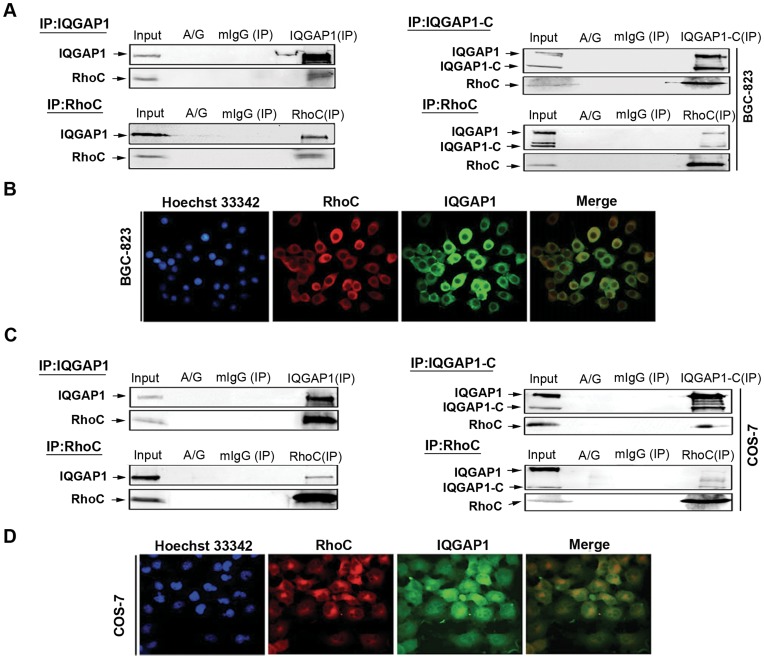
Identification of the interaction between RhoC and IQGAP1. (**A**) BGC-823 cells growing on 100 mm plates were transiently co-infected with Ad-IQGAP1 and Ad-RhoC-V14, or Ad-IQGAP1-C and Ad-RhoC-V14 for 48 h. The cells were lysed and equal amounts of lysate protein were immunoprecipitated (IP) with anti-RhoC, anti-IQGAP1 antibodies or isotype-matched IgG. Whole cell lysate was used as a protein input control. (**B**) BGC-823 cells were transfected with above adenovirus for 24–48 h, and the co-localization of RhoC and IQGAP1 in cells were determined by Immunofluorescence microscopy using anti-RhoC and anti-IQGAP1 antibodies. Nuclei were stained by Hoechst 33342 (blue). (**C**) COS-7 cells were transiently co-infected with Ad-IQGAP1-C/Ad-IQGAP1 and Ad-RhoC-V14 for 48 h. The cells were undergoing the same Co-IP procedure described above. (**D**) COS-7 cells were transfected with above adenoviral vectors for 24–48 h, and the co-localization of RhoC and IQGAP1 in cells were shown by Immunofluorescence. The data are representative from three independent experiments with similar results.

### BrdU Incorporation Assay

The DNA synthesis rate was measured with BrdU incorporation by Immunofluorescence. The seeding amount of cells was adjusted to attain a density of 70–80% confluence on the day of transfection. The plasmid pFlag-IQGAP1, pFlag-IQGAP1-C or pFlag-IQGAP1-N was co-transfected with GFP plasmid into BGC-823 cells using Lipofectamin™ 2000 as described above. 24 h after transfection, the cells were serum-starved overnight, treated with 200 *µ*M BrdU and incubated for about 16 h. The cells were then washed with PBS, fixed in freshly prepared 4%(v/v) paraformaldehyde at RT for 10 min, and permeabilized with 0.5%(v/v) Triton X-100, followed by incubation with DNase I (0.5 U/µl) for 30 minutes at 37°C. The cells were incubated with primary antibody against BrdU overnight at 4°C followed by Cy3-conjugated secondary antibody for 1 h at RT. Finally, nuclei were counter-stained with Hoechst 33342 for 15 minutes, rinsed with PBS for three times and visualized under fluorescent microscopy. Each assay was performed in quadruplet and repeated three times.

**Figure 6:**
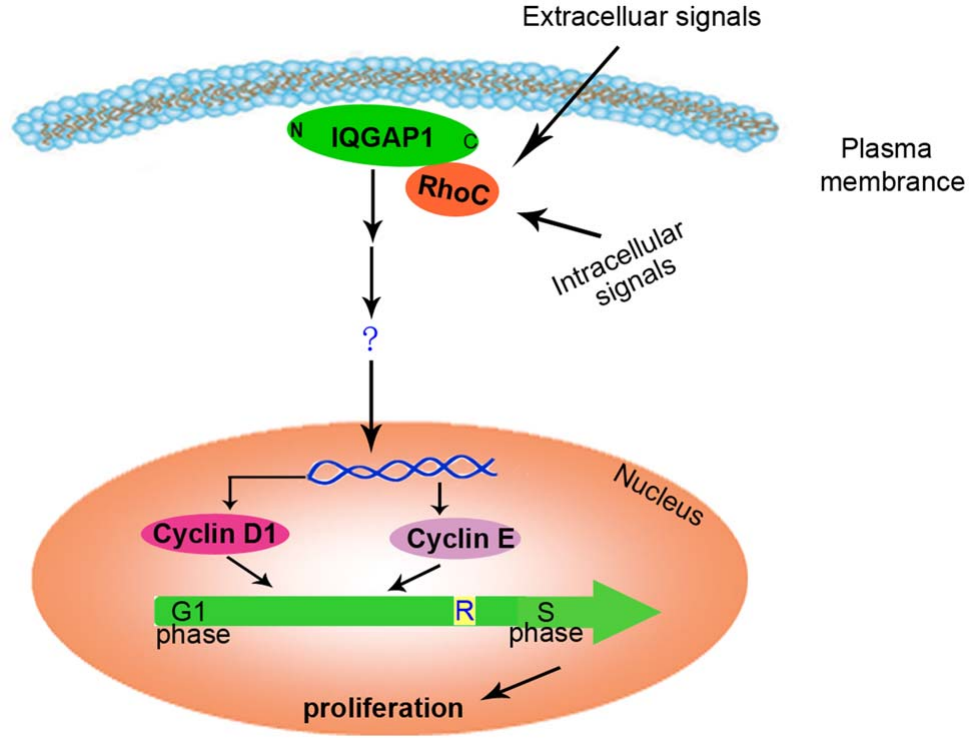
The schematic diagram of crosstalk between RhoC and IQGAP1 in gastric cancer. When RhoC is stimulated by extracelluar or intracelluar signals, it binds with the scaffold protein IQGAP1, influences the expression of cell cycle-related proteins such as cyclin D1 and cyclin B, affects G1-S transitions in the cell cycle, and then causes the change in cell proliferation. The signal transduction event through which IQGAP1 affects the expression of cyclin still needs to be elucidated.

### Co-immunoprecipitation

To investigate the interaction between RhoC and IQGAP1, COS-7 and BGC-823 cells were co-infected with Ad-IQGAP1 or Ad-IQGAP1-C, and Ad-RhoC-V14 for 48 h and then were lysed with RIPA buffer as described. The antibody against RhoC, IQGAP1 or isotype matched IgG was used for immunoprecipitation, respectively. Immunoprecipitates were analyzed by Western blotting as above, using anti-RhoC or anti-IQGAP1 antibody.

### Immunofluorescence Microscopy

The cells cultured on cover slips were fixed with freshly prepared 4%(v/v) paraformaldehyde in PBS for 15 min, permeabilized with 0.3%Triton X-100 in PBS for 10 min, and blocked with 3%(w/v) bovine serum albumin (BSA) in PBS. For Immunofluorescence, the cells on cover slips were sequentially incubated with rabbit polyclonal antibody against IQGAP1 at 4°C overnight, FITC-conjugated goat anti-rabbit IgG for 1 h at RT, mouse monoclonal antibody against RhoC for 2 h at RT, and finally goat anti-mouse IgG conjugated with Cy3 for 1 h at RT. Three washes were performed after each antibody incubation. The nuclei were counter-stained with Hoechst 33342. The result was observed under a fluorescence microscope assembled with a CCD camera (Leica).

### Statistical Analysis

The data were analyzed using a two-tailed ANOVA or Student’s *t*-test with SPSS statistical software, and expressed as means±standard deviation (SD). *P*<0.05 was considered significant.

## Results

### RhoC Promoted the Proliferation of BGC-823 Cells

The effect of RhoC on cell proliferation was also investigated. BGC-823 cells were infected with adenovirus encoding constitutively active RhoC and cell proliferation was detected by MTT assay. The results showed that RhoC also promoted the proliferation of gastric cancer cells BGC-823. Over-expression of RhoC-V14 in BGC-823 cells caused 48.7% increase in cell proliferation (Fig. 1A and B). Meanwhile, depletion of RhoC by siRNA reduced the proliferation of BGC-823 cell by 43.1% (Fig. 1C and D).

### IQGAP1 Enhanced the Proliferation of BGC-823 Cells

#### (1) Results of MTT assay

To assess the contribution of IQGAP1 to proliferation, MTT assay was used to detect the viability of gastric cancer cell line BGC-823. The results showed that compared with control cells (Ad-LacZ group), high expression of the C-terminal fragment of IQGAP1 (Ad-IQGAP1-C group) or full length IQGAP1 (Ad-IQGAP1 group) enhanced cell proliferation by 116% and 39%, respectively (*P<0.05, *P<0.05, Fig. 2A and B). In contrast, the N-terminal fragment of IQGAP1 has no obvious effect on the cell proliferation. On the other hand, interference of IQGAP1 expression with siRNA reduced cell proliferation by 43%, compared with negative control (*P<0.05, Fig. 2C and D). These results indicated that IQGAP1 was able to accelerate cell proliferation; the active domain was located in the C-terminal fragment of the protein.

#### (2) Results of BrdU incorporation assay

The BrdU incorporation assay yielded a pattern of response similar to that observed in MTT assay. Expression of Flag-IQGAP1 and Flag-IQGAP1-C markedly promoted DNA synthesis to the levels almost 1-flod and 2-fold than that observed with Flag-D1 (*P<0.05, *P<0.05, Fig. 2E and F), while IQGAP1-N did not shown any effects on the DNA synthesis, suggesting that IQGAP1 differently regulates DNA synthesis via different domains.

### Association between RhoC and IQGAP1 in Stimulating Proliferation of BGC-823 Cells

#### (1) RhoC and IQGAP1 did not affect the expression each other

The above results strongly suggest that both RhoC and IQGAP1 contributed to the proliferation of BGC-823 cells. In order to elucidate the possible association between RhoC and IQGAP1 in regulating cell proliferation, we firstly investigated if IQGAP1 and RhoC affect their expression each other. BGC-823 cells were transfected with siRNA to knockdown the expression of RhoC or IQGAP1, and then were infected with adenovirus encoding IQGAP1, IQGAP1-C or constitutively active RhoC respectively. Western blotting was applied to detect whether changing the expression and activity of one protein could affect the expression of the other protein or not. The results showed that increasing exogenous or knockdown of endogenous IQGAP1 or IQGAP1-C, did not affect the RhoC expression. Similarly, increasing constitutively active RhoC or interference of endogenous RhoC did not affect the expression of IQGAP1 or IQGAP1-C (Fig. 3A).

#### (2) IQGAP1 was an effector of RhoC in stimulating proliferation of the cells

MTT assay was used to elucidate the relation between RhoC and IQGAP1 in stimulating proliferation. The results showed that RhoC-siRNA had not obvious effect on proliferation caused by IQGAP1 (**P<0.01,Fig. 3B). However, depletion of IQGAP1 expression by siRNA blocked the proliferation caused by expression of constitutively active RhoC (*P<0.05, **P<0.01, Fig. 3C). This indicated that during the process of stimulating proliferation of BGC-823 cells, RhoC required the participation of IQGAP1. Furthermore, since RhoC’s effect required IQGAP1 while IQGAP1’s effect did not require RhoC, RhoC might be the upstream of IQGAP1 and took IQGAP1 as an effector.

### Effect of RhoC and IQGAP1 on the Expression of Cell Cycle Related Proteins

To further confirm the proliferation stimulating effect of RhoC and IQGAP1 and investigate the possible mechanism through which these proteins regulate the proliferation of the cells, we applied Western blotting to detect the expression of cell cycle related proteins in cells treated with methods of increasing or decreasing RhoC and IQGAP1. The results showed that the increase of RhoC and IQGAP1 stimulated the expression of cyclin E and cyclin D1, while it had no effect of the expression of cyclin B and CDK1/2. On the other hand, the decrease of RhoC and IQGAP1 inhibited the expression of cyclin E and cyclin D1. Meanwhile, in the case of RhoC silencing by siRNA, increasing IQGAP1 or IQGAP1-C still enhance the expression of cyclin E and cyclin D1 (Fig. 4). Theses results indicated that RhoC and IQGAP1 might regulate the proliferation through changing the expression of cell cycle related protein and causing more cells to enter S phase.

### RhoC and IQGAP1 Co-localized and Bound Each Other in Cells

Immunofluorescence and Co-immunoprecipitation method were applied to further examine the possible binding between RhoC and IQGAP1. Both BGC-823 and COS-7 cells were co-infected with Ad-IQGAP1 and Ad-RhoC-V14 or Ad-IQGAP1-C and Ad-RhoC-V14 for 48 h, and the total cell lysate was immunoprecipitated with anti-RhoC antibody and probed with antibody against IQGAP1, or immunoprecipitated with anti-IQGAP1 antibody and probed with antibody against RhoC, respectively. The results showed RhoC and IQGAP1 bound each other, with C-terminal fragment of IQGAP1 as the binding site with RhoC (Fig. 5A and C). Moreover, Immunofluorescence assay revealed that IQGAP1 was distributed mainly in the cytoplasm, where it co-localized with RhoC (Fig. 5B and D).

## Discussion

Uncontrolled cancer cell proliferation requires the coordinated and tightly regulated function of many proteins [23], [24], [25], [26]. It is, therefore, crucial to study the mechanism of how those proteins interact in regulating the cancer cell proliferation. Our previous results have documented that the expression of IQGAP1 and RhoC were increased in gastric cancer tissues and cancer cell lines [21]. The level of their expressions had a significant correlation with the poor differentiation of gastric cancer. However, it is not quite clear about whether IQGAP1 and RhoC are associated with cell proliferation in tumorigenensis. In this study, the expression level of IQGAP1 and RhoC and their biological activities in gastric cancer cell line BGC-823 were adjusted through infection with adenoviral constructs or transfection with plasmid DNA or siRNA. The proliferation of the cell was then investigated using BrdU incorporation assay and MTT method. The results showed that constitutively active RhoC significantly promoted the proliferation activity of gastric cancer cell line BGC-823, and depletion of RhoC by siRNA inhibited the traits. Research data have shown that RhoC had an important role in cell migration and metastasis [27], [28], [29], [30], [31], but there were some contradictions about whether RhoC regulate the process of transformation and proliferation in tumor cells [27], [29], [32]. Our results provide evidence that RhoC do have proliferation stimulating effect on gastric cancer cells. This will be helpful in elucidating the role of RhoC in regulating proliferation of cancer cells.

Our results showed that similar to RhoC, over-expression of exogenous IQGAP1 and IQGAP1-C also promoted proliferation of BGC-823 cells, whereas knockdown of endogenous IQGAP1 attenuated the proliferation ability of the cell, indicating that IQGAP1 also contributes to cell proliferation regulation. Considering that both RhoC and IQGAP1 have role in regulating proliferation of cancer cells and their expressions were highly correlated, we further explored the functional association between RhoC and IQGAP1. The results showed that in BGC-823 cells with knock-down of IQGAP1 expression, infection with adenovirus encoding constitutively active RhoC could not stimulate the proliferation activity anymore; while in BGC-823 cells with knock-down of RhoC expression, over-expression IQGAP1 or IQGAP1-C through infecting the cells with adenovirus encoding the corresponding DNA still caused higher proliferation activity. This indicated that RhoC drove tumor proliferation through IQGAP1, specifically through the C-terminal fragment of IQGAP1. This functional association between RhoC and IQGAP1 was further supported by Co-immunoprecipitation and Immunofluorescence. These results suggested that RhoC took IQGAP1 as an effector in regulating cancer cell proliferation, throwing new hint on the relationship of these proteins.

To primarily explore the downstream mechanism through which RhoC/IQGAP1 regulate the proliferation of gastric cancer cells, we investigated the effect of RhoC/IQGAP1 on the expression of cell cycle related proteins, including cyclin E, cyclin D1, cyclin B and cyclin dependent kinase (CDK). The results showed that both RhoC and IQGAP1 stimulated the expression of cyclin E and cyclin D1, but had no effect on the expression of cyclin B and CDK. The proliferation of eukaryotic cells is controlled at specific points in the cell cycle, particularly at the first gap phase (G1) to the DNA-synthetic phase (S) and the second gap phase (G2) to mitosis (M) transitions. Of which, the G1-S transitions is the major regulation point of the cell cycle. Cyclin D1 and cyclin E control the cell progression through promoting G1 to S phase of cell cycle. Our results suggested that when RhoC was activated by extracellular or intracellular signal, it bound to IQGAP1 and then influenced the expression of cell cycle-related proteins directly or indirectly through some unclear signal transduction steps, which could eventually lead to the change of cell progression and proliferation (Fig. 6).

In conclusion, these data from current study, in conjunction with previously published data [21], support the hypothesis that RhoC participates in both migration and proliferation of gastric cancer cells. IQGAP1 also participate the regulation of cancer cell proliferation as a downstream effector of RhoC. These data may shine lights on the development of therapeutic approaches for gastric cancer.
