# Correction: RhoC Regulates the Proliferation of Gastric Cancer Cells through Interaction with IQGAP1

**DOI:** 10.1371/annotation/84311f47-96d2-4b61-b36b-e1e8e3d8dc44

**Published:** 2013-06-14

**Authors:** Yan Wu, Yan Tao, Yongchang Chen, Wenrong Xu

1.) Figure 2 is missing text in panel E. Please see the correct version of Figure 2 at the following link: 

**Figure:**
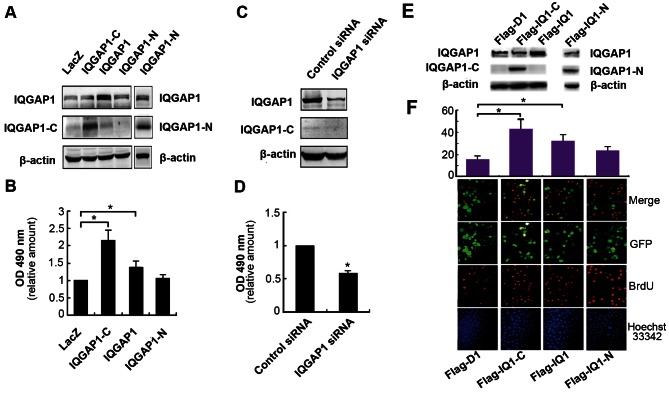


.

2.)The Reagents sub-section contains three typographical errors.

-In the text "The Rho C siRNA is a pool of", "Rho C" should read "RhoC".

-In the text "antisense 5’-UAGUU-CUCAA- AGACAGUAGtt-3’", "A- A" should read "AA".

-In the text "antisense 5’-UGUAUAAA-GUGCUGGUGUG- tt-3’", "G- tt" should read "Gtt".

